# PPPM health care - United States style: "political, profitable, and profligate medicine"

**DOI:** 10.1186/1878-5085-5-S1-A108

**Published:** 2014-02-11

**Authors:** Russell J Andrews

**Affiliations:** 1 Neurosurgery, Los Gatos, California, USA

## 

Health care in the United States costs at least 50% more per capita than in the European Union countries - upwards of 20% of the US Gross Domestic Product (GDP) - yet many measures indicate the health care outcomes in the US are inferior to countries spending much less. The reasons for this involve politics, profits, and wasteful practices that have evolved over decades.

The politics of US health care are clear in the Affordable Care Act (ACA or "Obamacare"). Both its history (following previous attempts at health care reform, e.g., by President Clinton in 1993) and its structure (nearly 1000 pages as Public Law 111-148 of the 111th Congress) are evidence of the "Balkanization" of health care which has evolved in the US. Perhaps only the US Tax Code exceeds the ACA in complexity and fragmentation - a testament to the influence of special interests (*aka* "lobbyists" representing insurance companies, hospitals, drug and device manufacturers, and health care professionals such as doctors) in the development of health care policy in the US. None of these groups has the interest of the patient (i.e., the health of the US populace) as its primary goal.

The profit motive has infiltrated all aspects of US health care. From reimbursement of doctors for the number of tests ordered and procedures performed (rather than for providing the most effective and efficient patient care), to financial incentives of the drug and device manufacturers to create new and expensive products (which are not necessarily better for the patient), to incentives of for-profit hospitals to provide inexpensive rather than effective care, to huge profit margins for health care insurers (whose management salaries, administration, and shareholders' profits may reach 30% of the premiums paid by the patient "consumer") - all aspects of US health care are guided by profits rather than the patient's welfare.

Wasteful practices in health care delivery abound in the US. Examples include the wasteful fragmentation evidenced by (1) the lack of a universal health care information system, (2) the multiple insurers (numbering in the hundreds in some regions), each with differing reimbursement policies and procedures, and (3) the proliferation of disposable devices (which usually provide a handsome profit margin for the manufacturers and marketers - often over 100%). The fact that a universal health care delivery system was "off the table" immediately in the initial health care reform discussions that led to ACA/Obamacare indicates that too many parties with substantial financial incentive to maintain the present inefficient but profitable (for the US health care industry) system were able to influence the politicians in Washington DC.

Ironically, Medicare (universal health care for people 65 years of age and older, implemented 50 years ago) is the one aspect in US health care delivery that no one wishes to change - but this fact is lost in the priority of profit over patient in the present reform of US health care. Other countries would do well to avoid the costly mistakes (costly in terms of both poor outcomes and high expense) being made in US health care delivery.

